# Digital Games and Mindfulness Apps: Comparison of Effects on Post Work Recovery

**DOI:** 10.2196/12853

**Published:** 2019-07-18

**Authors:** Emily Collins, Anna Cox, Caroline Wilcock, Geraint Sethu-Jones

**Affiliations:** 1 School of Management University of Bath Bath United Kingdom; 2 University College London Interaction Centre University College London London United Kingdom

**Keywords:** play, occupational health, mindfulness

## Abstract

**Background:**

Engagement in activities that promote the dissipation of work stress is essential for post work recovery and consequently for well-being. Previous research suggests that activities that are immersive, active, and engaging are especially effective at promoting recovery. Therefore, digital games may be able to promote recovery, but little is known about how they compare with other popular mobile activities, such as mindfulness apps that are specifically designed to support well-being.

**Objective:**

The aim of this study was to investigate and compare the effectiveness of a digital game and mindfulness app in promoting post work recovery, first in a laboratory setting and then in a field study.

**Methods:**

Study 1 was a laboratory experiment (n=45) in which participants’ need for recovery was induced by a work task, before undertaking 1 of 3 interventions: a digital game (*Block! Hexa Puzzle*), a mindfulness app (*Headspace*), or a nonmedia control with a fidget spinner (a physical toy). Recovery in the form of how energized participants felt (energetic arousal) was compared before and after the intervention and how recovered participants felt (recovery experience) was compared across the conditions. Study 2 was a field study with working professionals (n=20), for which participants either played the digital game or used the mindfulness app once they arrived home after work for a period of 5 working days. Measures of energetic arousal were taken before and after the intervention, and the recovery experience was measured after the intervention along with measures of enjoyment and job strain.

**Results:**

A 3×2 mixed analysis of variance identified that, in study 1, the digital game condition increased energetic arousal (indicative of improved recovery) whereas the other 2 conditions decreased energetic arousal (*F*_2,42_=3.76; *P*=.03). However, there were no differences between the conditions in recovery experience (*F*_2,42_=.01; *P*=.99). In study 2, multilevel model comparisons identified that neither the intervention nor day of the week had a significant main effect on how energized participants felt. However, for those in the digital game condition, daily recovery experience increased during the course of the study, whereas for those in the mindfulness condition, it decreased (*F*_1,18_=9.97; *P*=.01). Follow-up interviews with participants identified 3 core themes: *detachment and restoration*, *fluctuations and differences*, and *routine and scheduling*.

**Conclusions:**

This study suggests that digital games may be effective in promoting post work recovery in laboratory contexts (study 1) and in the real world, although the effect in this case may be cumulative rather than instant (study 2).

## Introduction

### Background

Workplaces demands on an individual’s physical, psychological, and emotional resources [1]. The need for rest and recovery exists across all employment environments, working patterns, and industries. Without sufficient recovery, negative strain and workload weigh on resources and can result in poor well-being, which can pose significant health risks over time [2,3]. Therefore, post work recovery (ie, the process of replenishing depleted resources after a day of work [2-4]) is vital in avoiding the physical and psychological health complaints associated with accumulated work stress [1,5]. In addition, successful recovery has been shown to have positive effects, for example, in encouraging positive affect [6,7] and encouraging greater work engagement, proactive behavior, and the pursuit of learning goals [8].

For recovery to be successful, 4 aspects need to be satisfied. These are psychological detachment (spending time not thinking about work), relaxation, mastery (the sense of gaining skills in something other than work), and control (the experience of having control within or over activities) [4]. Although the type of job itself plays an important part in recovery and work-related stress, leisure time is increasingly being appreciated as instrumental in this process [9]. Evidence suggests that mentally engaging leisure activities, for instance, are more effective than more passive activities [4]. Relaxing and socially oriented pastimes have been found to reduce work demands and negative affect during breaks [10], and activities such as sport or exercise have been highlighted as especially useful in improving recovery [11].

However, for many, leisure time recovery is difficult to achieve; the present-day workforce is working longer hours [12], traveling further to work [13], and reporting increased work stress compared with those of the past, all of which negatively impact this process. Consequently, some of the most effective activities, for example, team sports [11], are difficult to incorporate into our already busy days. One pursuit that is already well integrated into our home lives is the use of electronic devices. Therefore, although there are several factors that can impact the recovery experience itself, here we consider the role that digital technology can play in supporting it. We focus on the potential benefits of playing digital games and engaging with mindfulness apps and the role that the subjective experience of engaging in these activities might have. To this end, below, we outline the relevant literature regarding how these activities might impact recovery. Then, we describe 2 studies: Study 1 tests whether a digital game, mindfulness app, or no-activity control condition improves recovery after a work task in a laboratory setting. Study 2 takes an in-the-wild approach, aiming to more closely replicate how digital games and mindfulness apps might be used in real-world contexts. Study 2 therefore surveys workers over 5 days during which they either played a digital game or engaged with a mindfulness app after returning from work.

### Related Studies

Understanding whether digital activities can be used to contribute to the process of recovery not only indicates whether low effort, easily accessible activities can be used for this purpose but also whether the wide range of people currently using devices in their spare time stand to benefit. Evidence suggests that not all media use is equal in terms of recovery outcomes. For example, research has shown that watching movie clips is more effective than a no-activity condition in terms of recovery experiences [14], with positively valenced movie clips also improving relaxation [15]. The degree of interactivity of the media also appears to be influential. Digital games have been argued to satisfy all 4 aspects of recovery: high interactivity and immersion allow for psychological detachment, games tend to be relaxing, players can control progress within games, and games provide opportunities for mastery and accomplishments [16]. Surveys have indicated that those who play games for recovery purposes are more likely to play them after a stressful work situation, with people experiencing higher levels of work fatigue playing more games [16]. Similarly, in workplace contexts, game use during work time has been associated with greater reported recovery from work-related fatigue [17]. Those who regularly play digital games have also been found to have a lower need for recovery than those who do not [18], with particularly strong associations between gameplay and both relaxation and psychological detachment.

However, there have been few attempts to confirm that this relationship is causal. In 1 laboratory study, Reinecke et al [19] found that following a work task, playing a digital game improved recovery to a greater degree than watching a noninteractive movie clip or a no-activity control. This provides some promising initial evidence for a causal role of digital games in post work recovery, but further replications are required, particularly in more naturalistic settings.

There are also other smartphone apps that remain relatively unexplored in terms of recovery, such as those for mindfulness. Despite the rise in interest in mindfulness practices [20], the proliferation of mindfulness apps [21,22] and the promises many of these apps make in terms of well-being–related outcomes [22], little research has focused specifically on recovery. However, there are indications that it might be beneficial in this area. For example, mindfulness involves a state of attention and awareness of both external and internal states and experiences without any attributed judgment or value, promoting a sense of being in the moment [23]. It is this nonjudgmental experiential processing that has been argued to be effective against negative thought patterns such as rumination or anxiety, resulting in positive psychological effects and overall well-being [24], although the extent to which this extends beyond a placebo effect has been questioned [25].

Being equipped with the skills to prevent the negative effects of overthinking or ruminating on negative events has clear applications to occupational contexts, and mindfulness practice has been argued to be related to a number of factors relevant to post work recovery, including greater work engagement [26], improved sleep quality [27-29], and reduced emotional exhaustion [30]. In terms of recovery, more specifically, relaxation is one of the primary goals of mindfulness practice, and an in-the-wild study on work-life balance found that recovery in terms of psychological detachment improved with the implementation of mindfulness activities [31]. However, positive results have not been universal; in a field experiment, despite finding that mindfulness training improved sleep quality, there was no effect on psychological detachment [27]. Mastery and control have not been directly explored in relation to mindfulness, but it is plausible, for instance, that the reflective attention and awareness involved in mindfulness might provide a sense of control over one’s feelings or experiences and that improving one’s ability to practice mindfulness might provide a sense of mastery. However, whether this is indeed the case is unclear.

There has also been little research into how mindfulness practice in the format of an app specifically affects recovery. However, mindfulness apps have been associated with increases in positive affect, particularly when the task was enjoyed [32], and Web-based delivery of mindfulness programs has performed as well as in-person equivalents in improving stress, sleep quality, and heart rate [29]. Therefore, although existing evidence suggests mindfulness apps have positive effects that might translate into recovery outcomes, this is yet to be confirmed, particularly in comparison with interactive media such as digital games.

Another important factor to consider is the subjective experience of the activity; an activity 1 person might think of as a chore might be considered a respite by others, for example, cooking [33]. Similarly, an individual might appraise the same activity as either restorative or laborious depending on the context. The distinction between enjoyable and unenjoyable activities can be highly influential in the subsequent recovery outcomes, with those that are more pleasurable being more restorative [3,10]. This pattern has also been observed in research focusing specifically on media use. For example, negative perceptions of media use (such as believing it to simply be procrastination) restrict the extent to which such activities contribute to recovery [34,35], and enjoyment of the media activity (including playing digital games and watching movie clips) has been found to correlate with recovery as well as to mediate the relationship between recovery and energetic arousal [19].

### Our Studies

We present 2 studies that explore the impact of digital games and mindfulness apps on post work recovery (as measured by energetic arousal and recovery experience) and the role of enjoyment. Study 1 describes a laboratory experiment that compared the effect of a digital game, mindfulness app, and nonmedia control on recovery experience and energetic arousal following a work strain–inducing task.

Study 2 was an in-the-wild field study, taking place over a 5-day period in which workers either played a digital game or used a mindfulness app after work, again comparing changes in energetic arousal and differences between the groups in recovery experience. Enjoyment of the activity was also measured in both studies.

## Methods

### Study 1: Laboratory Study

Study 1 aimed to test the following hypotheses:

H1: The use of a digital game following a work task will be associated with a greater improvement in recovery (as measured by energetic arousal) and higher recovery experience than a mindfulness app that, in turn, will be associated with greater recovery than a nonactivity condition.

H2: Enjoyment of the activity will be associated with improved recovery.

#### Participants

A total of 45 participants (26 female) aged 19 to 36 years were recruited, all were students at a UK university. Participants were recruited through word-of-mouth and flyers on campus and at student accommodation and were entered into a prize draw to win £25 Amazon vouchers.

#### Design

The study was a mixed-design laboratory-based experiment exploring the change in recovery (inferred by energetic arousal scores) before and after taking part in 1 of the 3 break time activities (a digital game, mindfulness app, or nonmedia activity) following a work task aimed at inducing a need for recovery. In the nonmedia activity control condition, participants were told they had no activity but were provided with a fidget spinner for use at their own discretion. An additional dependent variable was recovery experience, measured after the break activity was completed.

#### Materials

##### Work Task

The work task was intended to create a need for recovery. On the basis of previous successful attempts at inducing work stress [36,37], the task involved a series of mathematical equations that were delivered and completed in an interactive PowerPoint presentation. Participants completed 10 arithmetic problems, shown as a sequential series of numbers, which took 15 min.

##### Break Tasks

In the digital game condition, the participants played *Block! Hexa Puzzle,* a digital puzzle game. This was selected as it is an easy game to play regardless of the participant’s previous experience, which has been identified as an important factor when testing digital games in terms of recovery [19].

In the mindfulness app condition, participants followed a 10-min mindfulness exercise from the app named *Headspace*. This app was again selected for its simplicity and clear instructions, meaning that participants could undertake the activity regardless of experience.

For the control condition, there was no designated media activity for the participant. In previous laboratory experiments on the effect of media on recovery, nonmedia control conditions involved sitting in a room and resting [15,19]. However, this could feel artificial or be boring for the participant and, thus, could impact recovery. Therefore, a toy called a fidget spinner was placed on the desk that the participant could use at their discretion.

##### Questionnaire Measures

The Activation-Deactivation Adjective Checklist (ADACL) [38] was used to measure energetic arousal as a proxy for recovery (as in the studies by Reinecke et al and Rieger et al [14,19,39]). This measure asks the participants to report to what degree they are feeling a series of emotional states, such as *energetic* and *tired*. Participants answered on a 4-point Likert-style scale. This measure was administered before the work task (T1), after the work task (T2), and after the intervention (T3), and it included 4 subscales: energy (mean alpha=.79), tiredness (mean alpha=.79), tenseness (mean alpha=.71), and calmness (mean alpha=.76). Energetic arousal was calculated by reverse scoring the tiredness subscale and summing this value with the energy subscale. Responses were not collected for 1 item of the ADACL (*drowsy*) because pilot testing suggesting this term was not understood and, therefore, to calculate the necessary scales, the responses for the most highly correlated item (*tired* [40]) were double weighted.

The recovery experience scale was used to measure recovery across the 4 recovery experiences [4], namely, psychological detachment (alpha=.83), relaxation (alpha=.92), control (alpha=.88), and mastery (alpha=.65). It contained 16 questions across the 4 dimensions for recovery, rephrased to refer to the activity as in the study by Reinecke [17] (eg, “When I [did the activity], I forgot about the work task”). The participants answered on a 5-point Likert-style scale.

Enjoyment was measured as in the previous study [19] with a 5-item scale, asking participants how much they agreed with statements such as, “[The activity] was fun” (alpha=.90). Participants answered on a 5-point Likert scale.

Basic demographic information was collected in the final questionnaire.

#### Procedure

The participants were asked if they used mobile games or mindfulness apps on their phone and were allocated to the condition they had no experience to avoid a confounding effect of previous experience. If participants had no experience of either, they were randomly allocated to a condition.

The first ADACL measure (T1) was administered through Qualtrics, a Web-based survey service. Once this was completed, the participants were presented with the work task through PowerPoint in presentation mode, which took 15 min. This task aimed to ensure that all participants were experiencing a need for recovery that had the potential to be reduced by the interventions. The participants then proceeded to complete the second administration of the ADACL (T2).

For the game condition and the mindfulness app condition, the participants were given a smartphone with the activity preloaded. For the control, the participants were given a fidget spinner that they were told could be used at their discretion. The participants were told that the break activity intervention would take 10 min.

Once the break was over, the following T3 measures were taken: final administration of the ADACL scale, recovery experience questionnaire, enjoyment measures, and demographic information.

### Study 2: In-the-Wild Study

The findings from Study 1 indicated the need for an in-the-wild approach to explore how digital games and mindfulness apps might impact recovery in real-world contexts. Moreover, previous work has highlighted the importance of exploring recovery on a daily level because of common fluctuations in job demands and recovery needs [41]. Therefore, Study 2 aimed to investigate the effect of a digital game and a mindfulness app in a naturalistic setting over a 5-day period. On the basis of the existing literature and the results of Study 1, the following were hypothesized:

H1: Participants in the digital game condition would demonstrate a significant increase in energetic arousal after performing the activity, and this increase would be significantly greater than that in the mindfulness app condition.

H2: Participants in the digital game condition would report significantly higher daily recovery experience scores than those in the mindfulness app condition.

H3: Enjoyment will be related to recovery experience and the change in energetic arousal before and after the activity.

#### Participants

A total of 20 participants were recruited (12 female), aged 19 to 58 years. To be eligible, the participants needed to be professionals working full time (7.5 hours per day for a minimum of 4 days per week). The participants were recruited through word-of-mouth and social media and were paid with a £5 Amazon voucher. Ethical approval was provided by the University Ethical Approval Board.

#### Materials

##### Break Activities

The same break activities were used as in Study 1; *Block! Hexa Puzzle* as the digital game and *Headspace* as the mindfulness app. The game or the mindfulness app was installed on the participants’ personal smartphones. Participants in the mindfulness app condition were instructed to follow the free 5-day beginners’ program provided by Headspace.

##### Questionnaire Measures

The participants were initially asked for demographic information, and recovery was again measured before (T1) and after (T2) the break activity intervention by the energetic arousal scale of the ADACL, measuring the subscales of energy (mean alpha=.84), tiredness (mean alpha=.92), tenseness (mean alpha=.81), and calmness (mean alpha=.71). The energetic arousal score was again calculated by adding the energy subscale to the reverse-scored tiredness subscale.

Recovery was also measured after the break activity by the recovery experience questionnaire [4], including the 4 recovery experience subscales: psychological detachment (mean alpha=.85), relaxation (mean alpha=.84), mastery (mean alpha=.75), and control (mean alpha=.90). Enjoyment was also assessed with the same measures as the laboratory experiment (mean alpha=.83).

#### Procedure

The participants were first asked if they already played digital games or used mindfulness apps, and if so, how often. They were then assigned to the activity that they did not have experience in. This was to ensure a similar level of experience across participants in each condition. The participants received guidance on installing the apps, and how and when to use them on each day of the experiment.

On each of the 5 days, when first arriving at home, the participants completed the T1 survey (the first administration of the ADACL measure). A prompt was sent via email to remind the participants to do so at the time they reported to arrive home. The participants then undertook the break activity for 10 min before being prompted via email to complete the T2 survey, comprising the second ADACL and the recovery experience measures.

When all 5 days of the activity had been completed, a semistructured interview was held over the phone or on the Web to understand the participants’ experiences. The interviews took approximately 10 to 15 min.

## Results

### Study 1: Laboratory Study

#### Manipulation Check

To identify whether the work task successfully created an additional need for recovery, energetic arousal at T1 and T2 was compared. A data collection error meant that T1 data were not available for 4 participants, so the analysis was conducted on the remaining 41. The mean energetic arousal scores and SDs can be found in Table 1. A paired-samples *t* test found no significant differences between energetic arousal scores at the 2 time points (*t*_40_=−.037; *P=*.97), indicating that the work task had not succeeded in creating an additional need for recovery. However, examination of the scores suggests that our participants started with lower energetic arousal than those in previous studies (eg, Reinecke et al [19] report prework task scores between 26.11 and 27.92) and that the T2 scores observed were similar to those following a successful work task (Reinecke et al [19] report post work task scores between 23.66 and 26.72). This suggests that participants were starting this study with a greater need for recovery than the participants in other studies and also started the break activity with similar levels. There were also no significant differences between the 3 conditions at T2, meaning that participants in all conditions started the intervention with equivalent levels of recovery (*F*_2,42_=.218; *P=*.81).

#### Analyses

##### Hypothesis 1

The first hypothesis was that the use of a digital game following a work task would be associated with greater recovery than a mindfulness app, which, in turn, would be associated with greater recovery than the nonactivity condition. Recovery was measured by energetic arousal administered at T2 (after the work task) and T3 (after the break activity) and the recovery experience scores administered at T3. The analyses for each dependent variable were conducted separately.

**Figure 1:**
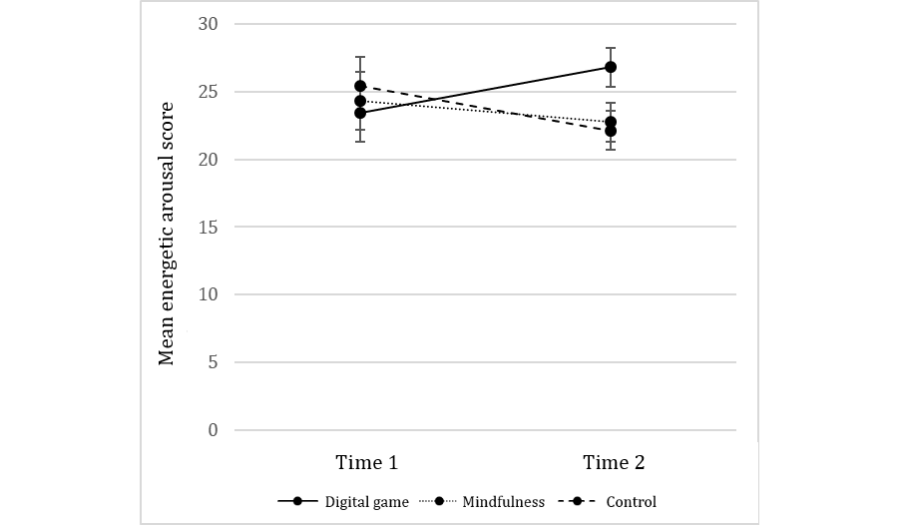
Mean energetic arousal scores for each of the 3 conditions at Time 2 (after the work task) and Time 3 (after the break activity).

Energetic arousal increased in the digital game condition between T2 and T3, with that of the other conditions decreasing between these time points (Figure 1). A 3×2 mixed analysis of variance (ANOVA) was conducted, with energetic arousal at T2 and T3 as within-subjects factors and condition as the between-subjects factor. There was no significant effect of condition (*F*_2,42_=.29; *P=*.75; *η*^2^=.01) and no overall effect of time (*F*_1,42_=.22; *P=*.64; *η*^2^=.01). However, there was a significant interaction between time and condition (*F*_2,42_=3.76; *P*=.03; *η*^2^=.15), suggesting the degree of change between T2 and T3 differed according to the condition. Follow-up post hoc analyses on the degree of change between T2 and T3 indicate that this significant interaction is mostly owing to the differences between the digital game and control conditions (*t*_28_=2.72; *P*=.01), and to a lesser extent, between the digital game and the mindfulness conditions (*t*_28_=2.04; *P*=.05). There were no differences between the mindfulness and control conditions (*t* 28=0.62; *P=*.54).

**Figure 2:**
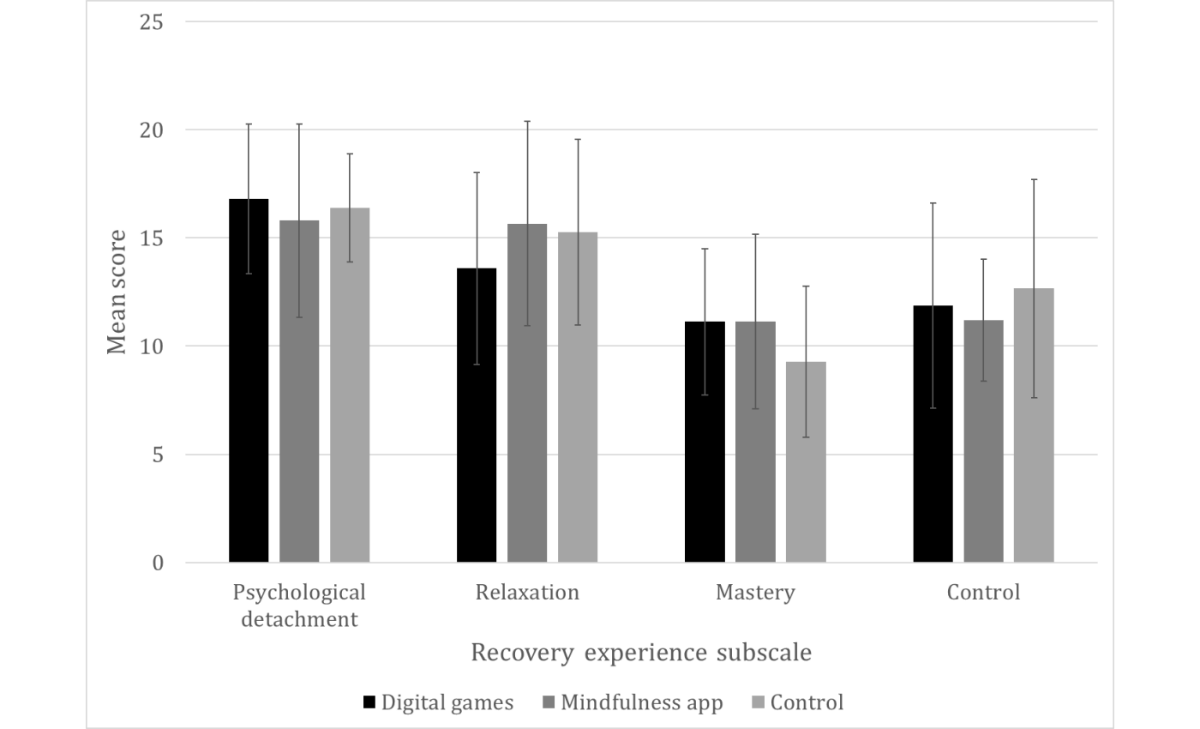
Mean scores for the recovery experience subscales across the 3 conditions.

A 1-way ANOVA was used to investigate the differences in recovery experience subscales (all taken at T3) and total score across the conditions. No significant differences between the conditions were identified (see Table 2 and Figure 2).

##### Hypothesis 2

**Figure 3:**
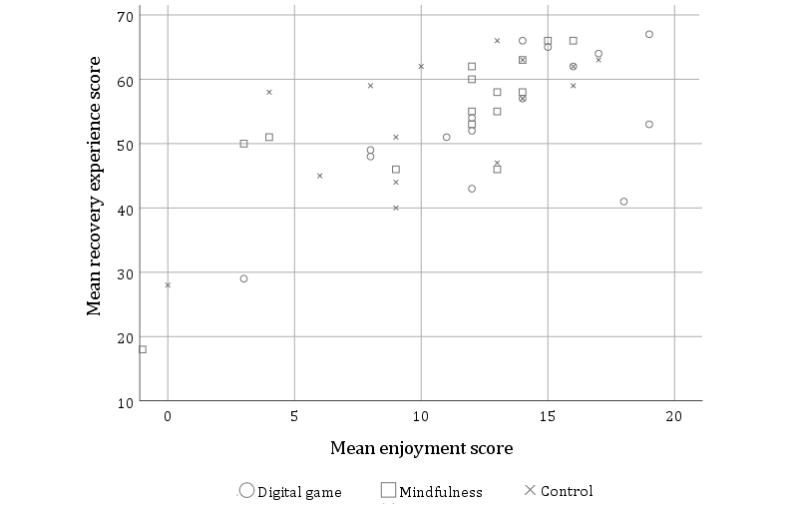
Scatterplot showing the correlation between enjoyment of the activity and Recovery Experience scores.

The second hypothesis was that the enjoyment of the activity would be associated with improved recovery. Although there were no differences between the conditions in enjoyment ratings (*F*_2,42_=1.47; *P=*.24), a Pearson correlation identified that enjoyment was significantly, positively correlated with recovery experience (*r*=.69; *P*<.001; see Figure 3), including all subscales except mastery (see Table 3).

**Table 1:** Mean energetic arousal scores (and SDs) across the 2 time points.

Condition	Time 1 (before work task), mean (SD)	Time 2 (after work task), mean (SD)
Digital game	26.15 (8.14)	23.40 (6.80)
Mindfulness	24.31 (5.68)	24.33 (9.15)
Control	23.93 (6.40)	25.40 (8.74)

**Table 2:** Results of a 1-way analysis of variance comparing the 3 conditions on recovery experience subscales and total score.

Measure	*F*	*η* ^2^	*P* value
Psychological detachment	0.30	.01	.74
Relaxation	0.90	.04	.42
Mastery	1.31	.06	.28
Control	0.43	.02	.65
Recovery experience total	0.01	.00	.99

However, the difference in energetic arousal between T2 and T3 was not correlated with enjoyment (*r*=.241; *P=*.11), indicating that although those who were more recovered rated the activity as more enjoyable, greater enjoyment did not result in an increase in recovery outcomes.

### Study 2: In-the-Wild Study

To retain the variations between the individual days within participants, and to examine the change over time, a model comparison approach [42,43] was used. Linear mixed-effects models were constructed with recovery measures (recovery experience and energetic arousal) as the dependent variables, condition and day as fixed effects, and participant as a random effect.

A total of 3 models were fitted for each dependent variable following Singer and Willett [44]. The first was an unconditional mean model, which operated as the control model. This model represents the null hypothesis that there is no change in the dependent variables over time and that there is no effect of condition. The second model was an unconditional growth model, representing the hypothesis that there is a change in the measure over time but that there is no effect of condition on the measure. By comparing the proportion of reduction of error (PRE) in this model with the PRE in model A, we can test if the measure is changing over time in days. Finally, the third model was a conditional growth model, representing the hypothesis that there is a change in the measure over time, and that there is an effect of condition on measure. Comparing this model with model B tests if the growth of the measure is affected by the data condition.

All models used ordinary least squares regression as this is the most parsimonious [44]. Maximum likelihood estimation was used because of the models having different numbers of fixed-effect terms [44,45].

#### Hypothesis 1

The first hypothesis was that participants in the digital game condition would demonstrate a significant increase in energetic arousal after performing the activity and that this increase would be significantly greater than that in the mindfulness app condition.

**Table 3:** Correlation coefficients between reported enjoyment and recovery experience subscales.

Measure	Enjoyment	*P* value
Psychological detachment	.584	<.001
Relaxation	.635	<.001
Mastery	.215	.16
Control	.421	.004
Recovery experience total	.691	<.001

To investigate the potential impact of the break activity on energetic arousal, the 3 models were constructed with the change in energetic arousal between T1 and T2 as the dependent variable. The conditional growth model was not found to be a significantly better fit for the data than the unconditional growth or unconditional mean models, indicating that there was no significant relationship between energetic arousal scores and either condition or day of the study.

#### Hypothesis 2

The second hypothesis was that participants in the digital game condition would report significantly higher daily recovery experience scores than those in the mindfulness app condition. The conditional growth model had a lower Akaikike Information Criterion (AIC) than the unconditional growth model (λ_2_=9.2; *P*=.01), which, in turn, had a lower AIC than the unconditional mean model (λ_3_=21.74; *P*<.001), indicating that the conditional growth model was a significantly better fit for the data.

Sequential (type 2 sum of squares) *F* tests were performed on the conditional growth model, using the Satterthwaite approximations for degrees of freedom and sigma adjusted to provide more conservative Restricted Maximum Likelihood-like results. The main effect of condition was not significant (*F*_1,18_=1.93; *P=*.18) and the main effect of day was not significant (*F*_1,18_=.00; *P*=.97). However, there was a significant interaction between condition and day (*F*_1,18_=9.97; *P=*.01); the parameter estimates indicated that recovery experience scores increased over time in the digital game condition but decreased over time in the mindfulness app condition.

In line with study 1 and previous literature, we also included an analysis of the recovery experience subscales. The 3 models were constructed for each subscale, which was compared and then analyzed using sequential *F* tests, as before. The model comparisons indicated no significant relationships for the control or psychological detachment subscales, although the conditional growth model was a significantly better fit than the other models for the latter (λ_2_=6.10; *P*=.05), suggesting that the lack of relationships with condition and day maybe because of ceiling effects. However, the significant interaction between condition and recovery experience was evident in the mastery (*F*_1,18_=5.99; *P*=.02) and relaxation subscales (*F*_1,35_=14.32; *P*<.001). For the relaxation subscale, there was also a significant main effect of condition (*F*_1,25_=9.40; *P*=.01). Relaxation increased over time for the digital game condition and decreased for the mindfulness condition. In addition, being in the mindfulness condition conferred a mean of 3.85 units more than being in condition 1; those in the mindfulness condition started with higher relaxation scores than those in the digital game condition, but their relaxation scores dropped as that of those in the digital game conditions increased.

#### Hypothesis 3

The third hypothesis was that enjoyment will be related to recovery experience and the change in energetic arousal before and after the activity.

The average enjoyment score (averaged across the 5 days of the study) was positively correlated with the average recovery experience score (*r*=.596; *P*=.01), relaxation (*r*=.657; *P*=.002), and mastery (*r*=.612; *P*=.004) but not with psychological detachment (*r*=.323; *P=*.16) or control (*r*=.278; *P=*.24). It was also positively correlated with the change in energetic arousal before and after the activity (*r=*.515; *P*=.02).

**Figure 4:**
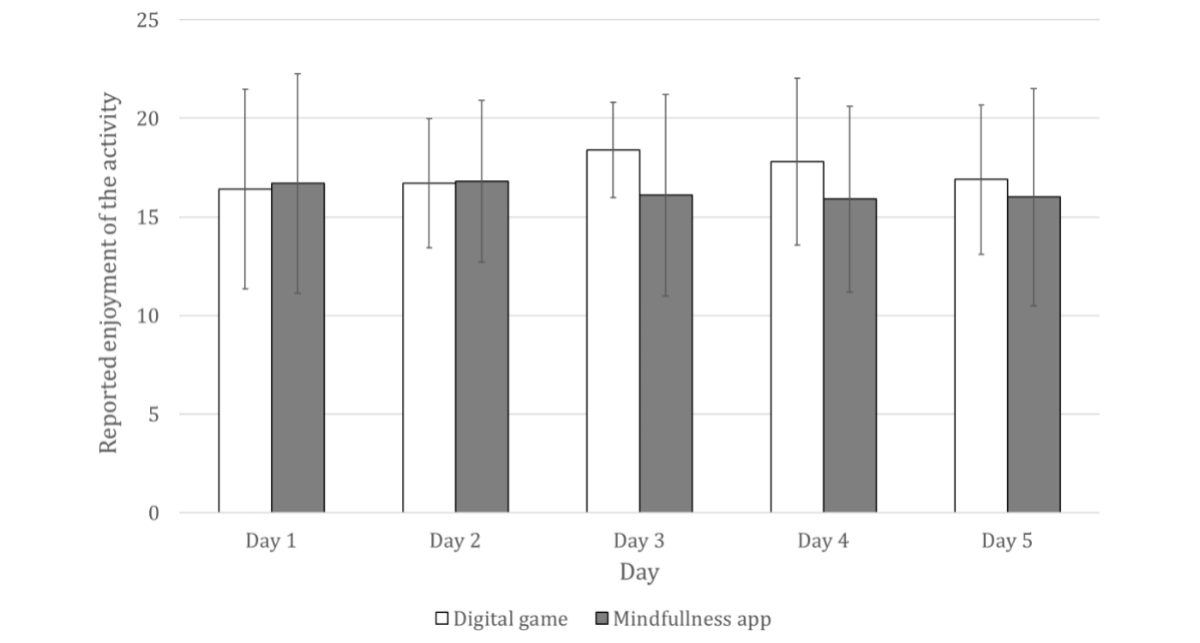
Reported enjoyment across the 5 days of the study and across the 2 conditions.

There were no significant differences between the conditions on either a daily level or in the average enjoyment score (see Figure 4). However, the digital game condition demonstrated a different pattern of correlations compared with the mindfulness condition. For instance, only in the digital game condition was the correlation between enjoyment and recovery experience significant (*r*=.738; *P*=.02), with no correlation in the mindfulness condition (*r*=.52; *P*=.13). In the digital game condition, there were also significant correlations between average enjoyment and psychological detachment (*r*=.672; *P*=.03) and mastery (*r=*.676; *P*=.03), whereas, in the mindfulness condition, there was only a significant correlation between enjoyment and the relaxation subscale (*r*=.799; *P*=.01). Similarly, only for digital games was there a significant association between enjoyment and the *change* in energetic arousal before and after the activity (*r*=.46; *P<*.01), with no such association in the mindfulness condition (*r*=.23; *P=*.11). This indicates that only in the digital game condition was it related to the *degree of change* in recovery as a result of taking part in the activity.

#### Qualitative Analysis

The interview transcripts were analyzed using thematic analysis [46] with a bottom-up approach. A total of 5 themes emerged, which were organized into 3 core themes: *detachment and restoration* (comprising of *detachment versus interruption* and *restoration and relaxation*)*, fluctuations and differences* (comprising *daily variations* and *personal preferences*), and *routine and scheduling*.

##### Detachment and Restoration

The *detachment and restoration* theme relates directly to the subjective experiences of recovery. The participants from the 2 conditions described their experiences in relation to detachment and restoration very differently.

This core theme contains 2 subthemes, *detachment versus interruption* and *restoration and relaxation*, combined because of the connection between the former as an experience and the latter as an outcome.

###### Detachment Versus Interruption

The 2 groups differed in the ways in which they discussed *detachment versus distraction*. For those in the digital game condition, part of the experience was a feeling of mental disengagement from the stresses of the workday, something that was referenced by all participants in this condition:

Even if you had a lot racing through your mind after a busy day at work, it was a good way to actually switch off from that, and once you'd finished the game, you felt as if you were actually relaxed and actually out of that work zone.

P2

This appeared to help participants in this condition to transition between work and home contexts:

I felt like I was restarted, you know, like a computer… It was such a short period of time, and then I felt very refreshed, and then I didn't think about work for the whole evening.

P20

Although this experience was not universal (P7 reported only minimal distraction with the digital game), it was in contrast to those in the mindfulness app condition. Only 1 of the mindfulness app participants mentioned renewed focus postactivity:

It's like okay, you've put that work behind you, but now you can get on with what other stuff you want to do, so it is that divide.

P3

When detachment was mentioned by those in the mindfulness condition, it was more in terms of physically taking time away from other activities and not as a psychological process:

It was taking time out I suppose. I guess it's a bit like going to have a facial or something.

P5

###### Restoration and Relaxation

A related theme that emerged from the data was *restoration and relaxation*, as many participants described their feelings after the activity in terms of arousal and energy. Participants in the digital game condition reported mixed outcomes. For example, 1 participant described feeling more relaxed after playing the game but also more tired (P11). A number of participants reported that they found the game calming, even if they did not enjoy the activity:

It was calming in a way, like I think because it's quite a monotonous type of activity.

P9

Conversely, other participants reported feeling more energetic after the digital game:

When you're tired from work, it just gets you a bit more alert.

P15

Within the mindfulness app condition, a number of participants described feelings of calm and relaxation but also sometimes tiredness:

I'd say all days I was more tired. But, definitely a few days it made me feel calmer. It was on like a scale.

P6

Other participants appreciated the tiredness that the mindfulness app encouraged as it helped with sleeping or bringing down energy levels when they were unwelcomingly high:

Sometimes if I have like a late football match and I get back late, sometimes I've still got the adrenaline going all over my body before I go to bed, and it takes a while to go to sleep. So, I think [the mindfulness app] kind of did help as well, so that was nice.

P8

##### Variations

The second core theme that emerged was related to variations; the participants were aware that the experience of performing both of the activities was not uniform, and the events of the day, moods, and personal preferences could impact their outcomes. This theme incorporates 2 subthemes: *daily differences* and *personal preferences.*

###### Daily Differences

Supporting the day-level approach of the quantitative analysis, 9 participants commented on daily variations in their experience of the activities. Several participants in both conditions commented that they found the activity more beneficial when they returned from a busier or more stressful day at work than on more calm days:

It depended on the day I'd had at work…I felt on the days that work was very busy I got a lot more out of the game.

P2, digital game

If [the day] was more stressed, I might have been slightly more resistant to relax, but it definitely helped…probably helps more on the stressful days.

P3, mindfulness app

However, there were exceptions with 2 participants who used the mindfulness app. For example, P6 reported that they found the activity more calming after a less stressful workday.

###### Personal Preference

There was also an appreciation that the effectiveness of the activities was not only dependent on the daily variations but also on differences in personal preference. Several participants reported that they did not enjoy the game (“I'm not really a gamey person.” [P12]), and they felt that this hindered potential benefits. The participants in the mindfulness app did not report a predisposition against mindfulness apps from the beginning as, often, this was their first time in trying the activity. However, through the study, some did learn that they did not enjoy using mindfulness apps.

Some participants mentioned that they felt they could get similar recovery benefits from other activities than those used in the study, choosing ones that they enjoyed more:

If I was going to do a game…it would be more kind of word based or something like that.

P12

Only participants in the mindfulness app condition reported that 10 min was too long for such an activity:

I think I would have enjoyed it if it was less time…I didn't enjoy it in the first place probably because it was so long, or it felt long.

P6

##### Scheduling and Routine

Some of the reported benefits of the activities were not so much to do with the game or app itself but more because of having a specific task scheduled for a particular time. For example, although participants said that after the activity, they “felt like doing something” (P15, digital game condition), further questioning indicated that it was more to do with the structure that the activity provided:

I think it was good to have a set routine when I got home that I had to do something instead of just kind of like sitting down and not really having a plan. It focussed me a little bit more, to know that when I got home, sat down, did the game, and then I moved on with the rest of my day then.

P11

Rather than continuing playing the digital game after the end of the study, 1 participant (P11) instead continued the routine, replacing the game with a walk, further emphasizing the role played by the schedule over the activity.

## Discussion

### Principal Findings

#### Study 1: Laboratory Study

Study 1 aimed to explore whether a digital game or a mindfulness app provided greater recovery outcomes than a no-activity control following a recovery-inducing work task. The digital game condition significantly improved energetic arousal between T2 and T3, with the mindfulness app and no-activity conditions showing a slight decrease. However, there were no significant differences between the conditions in the recovery experience. Enjoyment was positively correlated with recovery experience, but it was not related to the change in energetic arousal, suggesting that those who were more recovered reported the activity as more enjoyable, but the level of enjoyment itself did not impact recovery. A notable caveat is the lack of successful manipulation of recovery. One possibility is that this was because of the nature of the work task; although similar arithmetic tasks have been shown to induce work stress in previous studies [36,37], it has been argued that fast-paced tasks are more effective at reducing energetic arousal levels [47]. For instance, some previous work has instead used tasks such as highlighting specific letters in texts (eg, the study by Reinecke et al [19]). However, another explanation for the lack of an effect is that our participants had lower baseline energetic arousal than in previous studies, which meant that an additional reduction in these scores was not possible. This is supported by the discovery that the observed time 2 energetic arousal scores were comparable with those from studies in which the work task was successful in inducing a need for recovery, suggesting that participants were still starting the break activity with depleted resources. These relatively low levels of energetic arousal, coupled with the discovery that the effect of the digital game was in the opposite direction to that of the other conditions, indicate that the digital game may still be restorative in a manner that the other conditions are not. The lack of significant differences between the conditions in energetic arousal following the work task is also reassuring, as even with the potentially polarizing effect of the work task, participants in all 3 conditions were beginning the break activity with a similar overall level of energetic arousal.

This study also found no difference between the conditions in terms of recovery experience scores. Previous research suggests that digital games are more effective in promoting recovery than nonmedia or noninteractive activities and that the recovery experience measure tends to reflect the energetic arousal scores [14,15,19]. As the wording of the recovery experience measure emphasizes feeling differently following the break activity intervention, the score could have been impacted by the unsuccessful recovery manipulation to a greater degree than the energetic arousal measure, for which there is greater variation and less of a focus on comparative experiences. Taking these results with those of Study 2 which was able to avoid these issues is therefore important in gaining a full understanding of the impact of digital games and mindfulness apps on recovery.

#### Study 2: In-the-Wild Study

Study 2 aimed to explore the impact of a digital game and a mindfulness app in a real-world context over 5 days. It was predicted that those in the digital game condition would show a greater increase in energetic arousal scores following the activity compared with those in the mindfulness condition and that those in the digital game condition would also demonstrate higher recovery experience scores. Enjoyment was predicted to be related to both the final recovery experience scores and the change in energetic arousal between T1 and T2, indicating that enjoyment underpins the relationship between activities and recovery. Multilevel modeling identified that there were no differences between the conditions in terms of the degree of change in energetic or tense arousal before and after the activity. However, there was a difference between the 2 conditions in the pattern of recovery experience; in the mindfulness app condition, recovery experience scores (particularly relaxation and mastery) steadily decreased during the 5 days of the study, whereas in the game condition, they increased. Therefore, although there was no evidence of either activity impacting recovery on a daily level, there appeared to be a cumulative, positive effect of the digital game.

Follow-up interviews allowed further exploration of the subjective experiences of the activities, with the core themes of *detachment and restoration* (comprising *detachment versus interruption* and *restoration and relaxation*), *fluctuations and differences* (comprising *daily variations* and *personal preferences*), and finally, *routine and scheduling* emerging from the interview data. Differences between the conditions in opportunities for detachment from work were particularly relevant and shine some light on the quantitative findings concerning the cumulative effect of digital games on recovery experience scores.

The discovery that there was no daily effect of the activities on recovery, particularly in relation to the change in energetic arousal scores, runs somewhat counter to previous studies. Laboratory studies have shown that even after short work tasks, digital games can successfully impact energetic arousal scores to a greater degree than nonmedia activities [15,19]. The failure to replicate these findings may simply be because of the more complex and less controlled environment of in-the-wild studies, the less uniform experience of a day’s work in comparison with a specified work task, or the differences in timings between work and the completion of the intervention. Work stress and subsequent recovery needs have been argued to vary from day to day [41], making a clear-cut relationship between activities and recovery outcomes in the absence of any other influences unlikely. Nonwork leisure activities have been said to impact individuals on a daily level in a manner that prevents the buildup of work stress, which, in turn, manifests in poor health outcomes [41]. Therefore, the discovery of a cumulative effect, but not a daily effect, of digital games is not wholly surprising and is in accordance with much of the more general recovery literature.

Alternatively, the cumulative effect of the digital game could be because of the increases in gaming skills over the experimental period; previous research has found that greater gaming skill is positively related to the degree of mood repair experienced after playing a digital game [48]. This would suggest that the more our participants played the game, the more they improved, which in turn had a larger impact on their mood. However, it is difficult to know whether such an effect would occur for a simple game such as *Block! Hexa Puzzle.*

The negative pattern of recovery in the mindfulness app condition over the course of the study was particularly interesting and surprising. It is possible that this pattern is simply because of the need for recovery increasing during the working week; however, without a control group or baseline, it is not possible to conclude whether this decline in recovery was due to the mindfulness app negatively impacting recovery or whether this pattern would have occurred as a result of passing time regardless of our intervention.

The in-the-wild interviews indicated that personal preference may have had an impact on enjoyment, supporting previous research that identified that different people can evaluate the same activity as relaxing or as a chore [49] and that individual preference could result in whether a person enjoys and subsequently recovers from an activity [10,50]. Considering that previous research has also highlighted the role of personal appraisals of the activity (eg, how beneficial or worthy they are seen to be or how much they constitute procrastination rather than a legitimate way of spending time) in promoting recovery [16,19,34], this might be an interesting route for future research. Another interesting point raised was the role of the activity as a scheduled break point and the benefits that emerged because of this rather than the activity itself. This supports previous assertions that implementing specific routines can be beneficial for post work recovery [51].

##### Limitations

The first limitation of this study is that the nature of the study means that participants were trusted to follow instructions and perform the activities in the manner requested, but no checks were made to ensure this was the case. This is a risk associated with many in-the-wild field studies, and the only alternative would have been to install a software to track the activities performed on participants, which would have likely reduced interest in participating.

The second is that participants were not able to choose their activity and were instead pseudorandomly assigned to one of the conditions. Although this method helps avoid the influence of possible person-level confounds, enjoyment and the subjective experience of activities are known to be important factors in recovery outcomes [33]. Consequently, it is possible that allowing participants to choose the activity they would enjoy the most would have increased the observed restorative effect. However, this would have reduced confidence in our conclusion that any differences between the conditions were in fact because of the activity and not other related variables. The lack of extreme scores for the enjoyment measure and the lack of overall differences in the score between the conditions suggest that there were no participants who reacted negatively to the activity and that, generally, the activities were seen as equally as enjoyable. Future work may wish to explore the effect of personal choice of activity on recovery outcomes.

Finally, there is the possibility of a ceiling effect for psychological detachment that prevented us from exploring the possible impact of the activities on this measure. This was suggested by the model analysis, which found that although there was no significant association between the measure and the day of the study or the condition, the conditional growth model was a significantly better fit than the other models. Future studies may wish to specifically target individuals with low psychological detachment to explore how these activities impact this measure.

### General Discussion

This paper outlines 2 studies that aimed to explore whether a digital game and a mindfulness app were able to improve post work recovery. Study found that the digital game condition was the only one to significantly increase energetic arousal, although there were no differences between the conditions in terms of recovery experience. Study 2 found that although neither condition appeared to impact participants in terms of either energetic arousal or recovery experience on a daily level, those in the digital game condition demonstrated an accumulative effect on recovery experience, with scores gradually increasing over the 5 days.

The discovery that digital games have the potential to improve recovery above and beyond more passive activities is supported by previous research [14,18,19,35]. However, our support for this is somewhat tentative owing to the lack of differences between the conditions in terms of recovery experience in Study 1 (with the only differences being in relation to the degree of change in energetic arousal before and after the activity) and the lack of differences in energetic arousal in Study 2 (with differences only occurring in terms of recovery experience over time). Although both energetic arousal and recovery experience scores are intended to operate as proxies for recovery, they do so in very different ways; the energetic arousal score reflects the affective state associated with good recovery, whereas the recovery experience scale directly asks individuals to what degree the activity provides the 4 different recovery experiences. Therefore, although the strongest evidence for a positive role of digital games in recovery would be for the effect to be evident across both measures, it is not surprising to have different patterns in each. This is particularly the case considering that owing to the nature of the measures, this study focused on the change in energetic arousal and just a one-time score for recovery experience.

However, one clear conclusion across both studies is a lack of effect of the mindfulness app; the mindfulness app was not able to improve recovery outcomes in terms of energetic arousal or recovery experience scores, in the laboratory or in-the-wild. Previous research exploring the impacts of mindfulness practice in promoting psychological detachment [31] is mixed, with little other work conducted on the relationship with recovery more generally. Although it is possible that the use of mindfulness apps has positive outcomes in terms of well-being or positive affect, this study suggests no benefits in relation to recovery.

There was also a clear relationship between enjoyment and recovery. However, in Study 1, enjoyment did not correlate with the *change* in energetic arousal before and after the activity, suggesting that a more enjoyable activity did not result in greater increases in recovery. However, in Study 2, the average enjoyment rating correlated with both the average recovery experience score and the degree of change in energetic arousal before and after the activity, in line with previous research [19,32,52]. The lack of a successful manipulation of energetic arousal in Study 1 could be responsible for this discrepancy; without enough variance between pre- and postenergetic arousal scores, it is unlikely that enjoyment could improve energetic arousal to a great enough degree to result in significant correlation.

### General Limitations

In addition to the limitations discussed in the 2 individual studies, there were other overarching limitations that need to be acknowledged. The first was the reliance on self-report measures as proxies for recovery. Although such measures have been used successfully in previous studies exploring the effect of digital games [15,19], future work may wish to supplement these measures with cognitive tests that are able to identify whether participants also demonstrate evidence of being cognitively recovered through calculating error rates. This study has also highlighted other interesting avenues of investigation for future research. For example, Study 2 was not able to explore any differences in the effects of the activities depending on the nature of the participants’ work or the specific demands of their roles. Similarly, collecting baseline data would strengthen the conclusion that changes in recovery outcomes are attributable to the activities undertaken and not the progression of the week. Finally, measuring personal appraisals of digital game use and their perception as procrastination or a pastime would be a welcome addition to future work, considering the existing literature on how negative perceptions of media use hinder their effect on recovery [34,35].

### Conclusions

Together, these 2 studies suggest that digital games may be effective in promoting post work recovery in laboratory contexts, even without the depleting effect of a work task (Study 1) and in the real world, although the effect in this case may be cumulative rather than instant (Study 2). Our qualitative findings further highlight the roles of enjoyment and personal preference (suggesting that those who enjoy digital games may benefit the most), the daily changes in recovery needs (indicating that activities may impact differently depending on the demands of the day), and the scheduled nature of the activity (suggesting that having a specific time for playing digital games could be especially effective).

## Abbreviations

ADACL: activation-deactivation adjective checklist
AIC: Akaikike Information Criterion
ANOVA: analysis of variance
EPSRC: Engineering and Physical Sciences Research Council
PRE: proportion of reduction of error
